# Supplementary material for: Large-Scale Compatible Roll-to-Roll Coating of Paper Electrodes and Their Compatibility as Lithium-Ion Battery Anodes
Source: Nanomaterials (Basel). 2025 Jan 14;15(2):113. doi: 10.3390/nano15020113 (PMC11767952; doi:10.3390/nano15020113)
Supplement: Supplementary file 1 [file nanomaterials-15-00113-s001.zip › Supplementary- Large-scale/Supplementary__Large_scale.pdf]

Supplementary Materials: Large-scale compatible Roll-to-Roll coating of paper electrodes for energy storage applications

Nicklas Blomquist [1,*], Manisha Phadatare [1], Rohan Patil[1], Renyun Zhang [1], Noah Leuschen [1] and Magnus Hummelgård [1]

1. Electrode material preparation

The nanographite in Slurry B was fabricated in house by our earlier water-based large-scale compatible exfoliation technique [1]. For this study a 400 L nanographite suspension was prepared with 30 kg (75 gL^{-1}) thermally expanded natural crystalline graphite (EXG 9840) from Graphit Kropfmühl in Germany, 1200 g polyacrylic acid at 50 wt% from SIGMA-ALDRICH in USA and 369 L de-ionized water. The suspension was forced trough a 1 m long and 2 mm in tube diameter helical coil shear zone (referred to as S2 in the original method) ten times at a constant flow rate of 5 $Lmin^{-1}$ by a tree cylinder high pressure piston pump. Figure S1-A shows the high pressure pump and helical coil shear zone used during exfoliation. Figure S1-B shows the two processing containers, where suspension in container 1 is feed into the pump inlet for exfoliation and the shear zone outlet is connected to container 2. After one full pass the suspension in container 2 was transferred to container 1 and the process was repeated until 10 full passes. Figure S1-C shows the shear zone outlet in container 2 during the second pass.

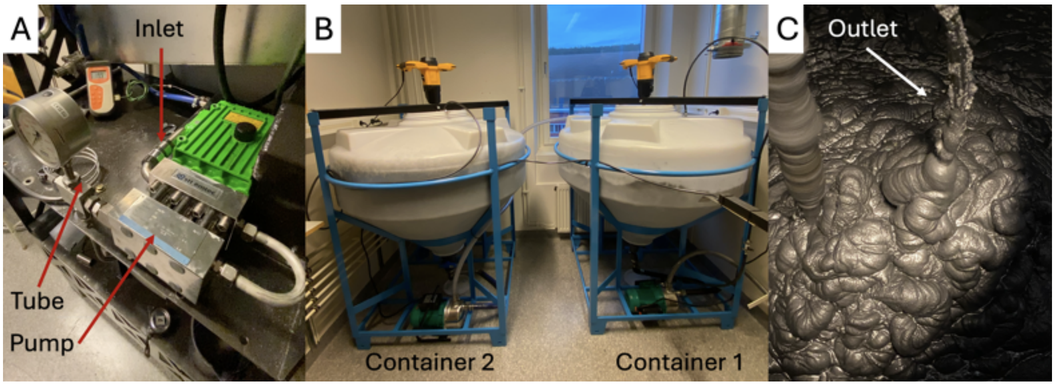

Figure S1. A) shows the high pressure piston pump connected to the helical coil tube shear zone. B) shows the two processing containers, one feeding the the exfoliation process with suspension and one retreving exfoliated material. C) shows the shear zone outlet in container 2 during the second full pass.

Prior to coating 3 kg Refined MCC (White MCC) from Fibenol in Estonia was added to the exfoliated nanographite suspension using a Cowles mixer to achieve a uniform coating color. The final slurry volume was 412 L and a solids content of 8 wt% (Slurry B).

2. Electrode coating and sample preparation

The coating was performed in collaboration with UMV Coating Systems at their Pilot Plant in Säffle Sweden. The applicator used was the UMV Liquid Application System (LAS), a hydrophilic roll coater that applies a pre-metered film of coating color to the substrate. The main coating parameters used in this study was Machine speed (paper speed), Transfer roll speed and Metering nip. The specific values of these settings are presented in the Results section in Table 1. Figure S2-A and -B shows the coated paper substrate exiting the coater applicator and entering the IR-drying section. Figure S2-C and -D shows sample cut-outs from Roll 08 and Roll 11 respectively. Figure S2-E shows three sample strips with a width of 3 cm cut across the full with from one of the rolls, -F shows the procedure of punching out 16 mm coins in sets of 100 from each sample strip. Figure S2-G and -H shows

simple weight and thickness measurements of the sample discs, respectively. Figure S2-I shows a a schematic sketch of the LIB half-cell assembly.

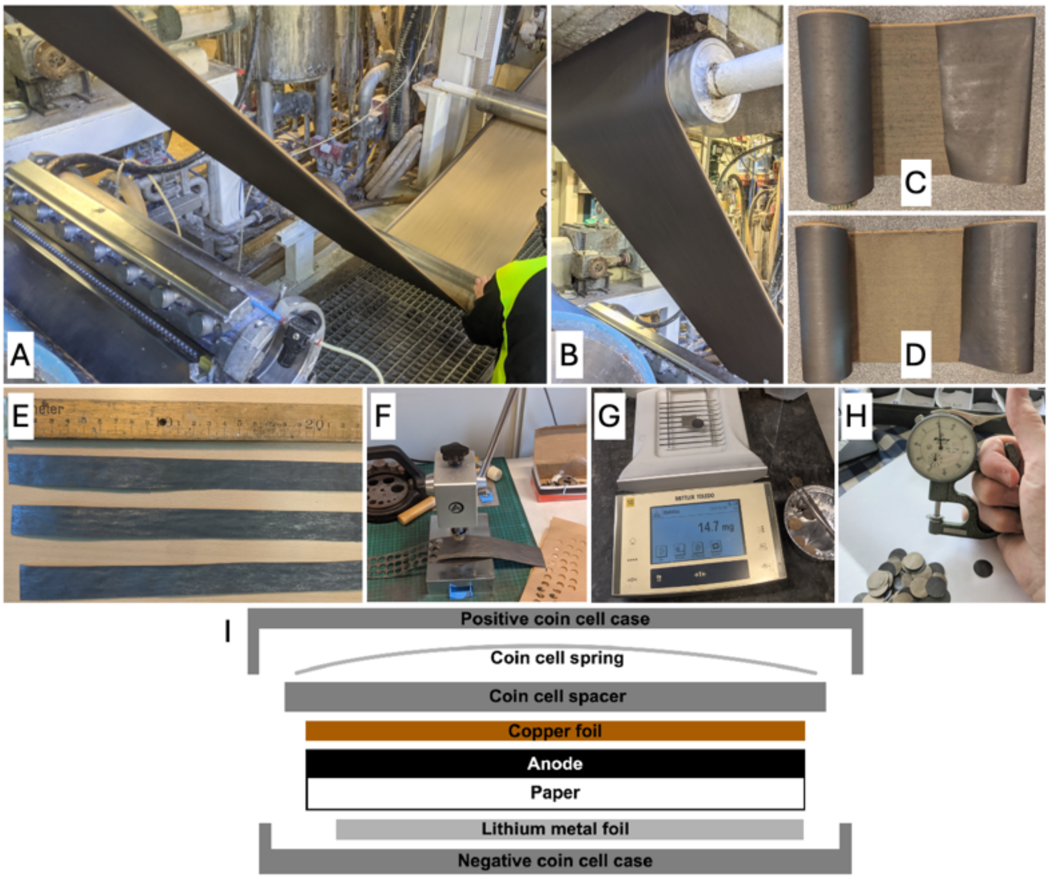

Figure S2. A) and B) shows the coated paper substrate exiting the coater applicator and entering the IR-drying section from two different angles. C) shows sample cut-outs from Roll 08 and D) from Roll 11. E) shows 3 cm sample strips cut across the full with from one of the rolls, F) the procedure och punching 16 mm coins from each sample strip, G) shows weight measurement of punched out sample disks, H) shows thickness measurement of the sample discs and I) a schematic sketch of the LIB half-cell assembly

3. Study of electrical dependency of humidity

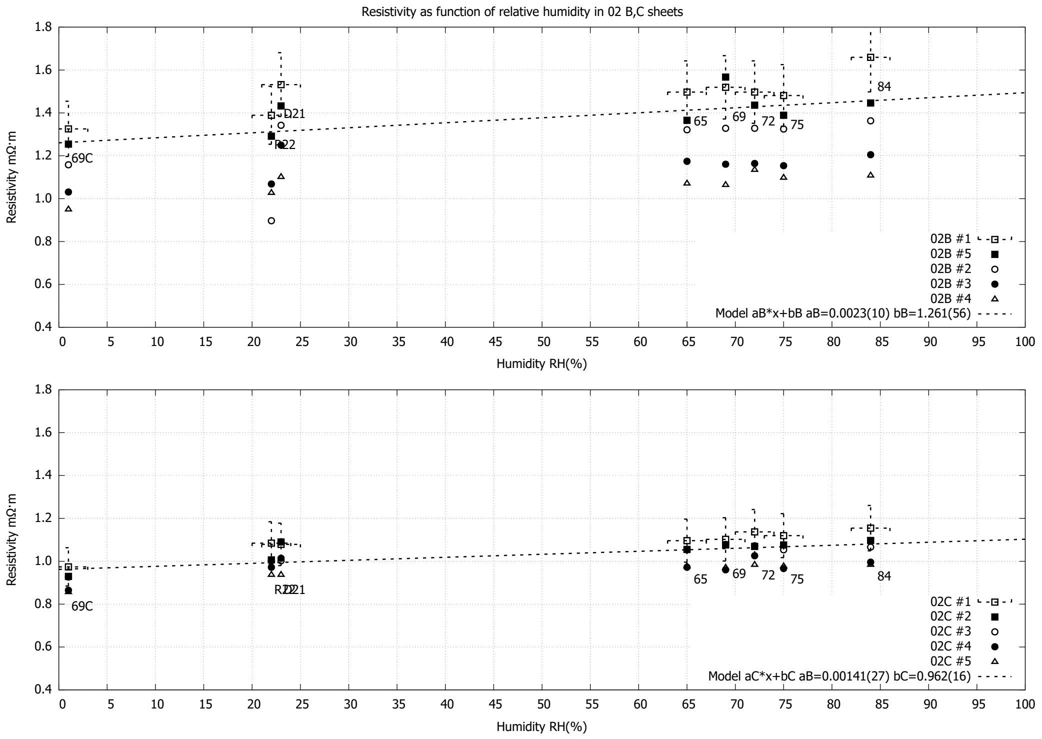

Figure S3. Humidity dependency tested for coated sheets 02B (top) and 02C (bottom). Both sheets 02B and 02C was made with Slurry A on Plus paper substrate. Precut A4 sample sheets from coating trials 02B and 02C was cut into five stripes 10 mm wide, 30 cm long and with guillotine. Room humidity was measured to be 22%RH followed by electrical resistance measurement with multimeter and this by placing the stripes on a plastic ruler with the electric connector-clamps at its ends. Each stripe were thereafter put into desiccator boxes with salt-humidifiers giving a fixed humidity of 65%RH, 69%RH, 72%RH, 75%RH, and 84%RH for 24h periods with followup resistance measurements. All samples were then in 24h intervals rotated between all the humidity options giving five data sets for each 02B and 02C sample. Experiment ended with a final treatment of all samples in oven to be dried over night at 69C. Results shows very small trend of increasing resistance with humidity but is insignificant if compared to the error estimates of data, (error bars only shown for top row of dataset for better clarity in graph).

4. Study of shelf-life dependency of resistivity

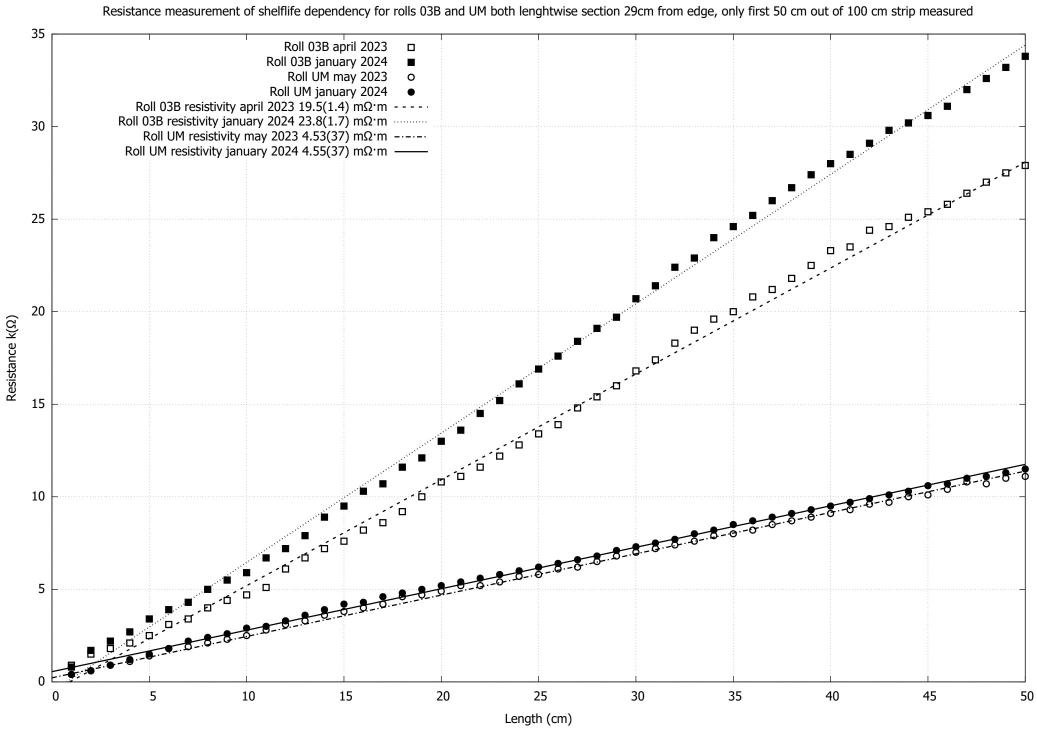

Figure S4. Electrical resistance was measured with multimeter in centimeter intervals over the length of stripes that were cut into lengthwise sections taken from roll 03B and UM. The measurement was repeated after 7-8 mounts passed. For roll 03B the resistivity increased by a minor 22% and for roll 11 no change was observed.

5. Study of influence of calendering on the coating thickness and electrical resistivity

Table S1. Two sheets ofA4 size were cut from each of the coated rolls, one sheet was calendered without heat, while the second sheet was left for comparison. Stripes about one inch width was cut out from the sheets in lengthwise direction of paper followed by thickness-, width-, and electrical-resistance measurements in similar way as described in method section. Calculation of coating compression was done as well as comparison of coating thickness reduction, density increase, and electrical resistivity decrease due to the calendering. Calendering effects were not studied on roll 03B and UM. *referees to uncoated papers hence weight, thickness, density, and compression ratio is for the full paper. †for roll 10 the coating was too uneven and when two samples were compared the individual variation between samples was larger than the effect of the calendering leading to a results in which the calendering sample appeared thicker than the uncalender version.

**Roll number**	**Coating weight [g/m^2]**	**Coating thickness [μm]**	**Cal. Coating thickness [μm]**	**Coating Comp. ratio**	**Coating density [g/cm^3]**	**Cal. Coating density [g/cm^3]**	**Resistivity [$m\Omega m$]**	**Cal. Resistivity [$m\Omega m$]**
Plus*	68.54(22)	96.60(50)	74.38(58)	23%	0.71	0.92	-	-
Boost*	78.87(19)	124.27(38)	88.39(26)	29%	0.63	0.89	-	-
06	8.39(33)	40.2(1.4)	24.00(90)	40%	0.21	0.35	3.689(55)	1.434(23)
07	11.42(31)	60.6(3.0)	21.38(85)	65%	0.19	0.53	2.702(54)	0.5593(69)
08	12.83(22)	26.10(85)	11.5(1.0)	56%	0.49	1.12	0.4222(58)	0.1293(17)
10	13.19(75)	52.2(1.3)	97.2(3.0)	-90%†	0.25	0.14	1.240(34)	4.47(23)
11	17.65(29)	73.3(2.6)	28.29(76)	61%	0.24	0.62	1.307(23)	0.3560(45)
12	10.79(24)	74.1(3.2)	45.6(1.1)	38%	0.15	0.24	10.88(30)	2.801(40)

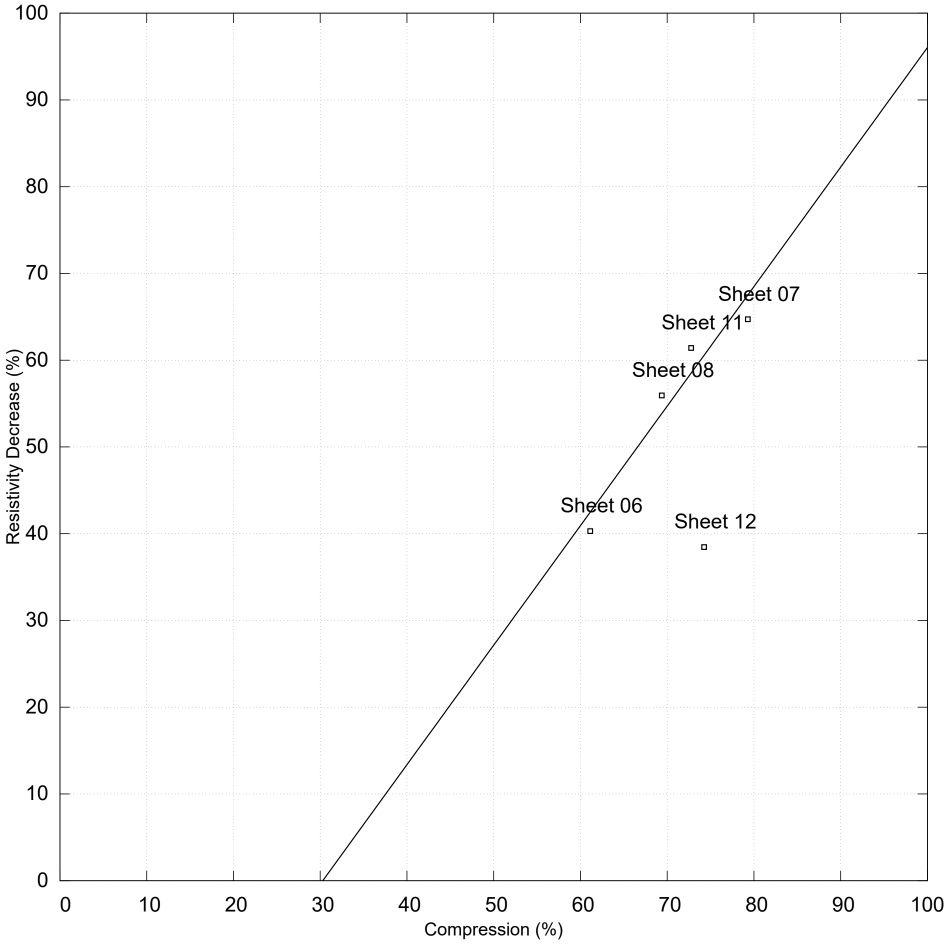

Figure S5. Comparison of resistivity decrease as function of compression of the coating due to calendering. Sheet 06 to 11 follows a linear behavior as expected for different compression ratios. However the change is not 1:1 i.e. if the compression is 60% as for sheet 06 a change in resistivity should also be 60% since resistivity depends linearly on thickness of coating but this is not observed, instead only 40% resistivity decrease is found and the ratio becomes 1.37 between resistivity decrease and compression. The regression gives an intercept of y-axis at -41.7 with is unrealistic. Sheet 12 was excluded from the regression calculations and is seen far of from the trend line, this can be explained by the achieved lower coating density of Sheet 12 is not caused by a uniform porosity increase but rather of uneven coating which is also confirmed in SEM imaging, see figure S10.

6. Complete data table of all measurements

Table S2. **A** sample, **B** machine-speed(m/min), **C** transferroll speed(m(min), **D** coating thickness(um), **De** thickness error(um), **E** coat weight(g/m^2), **Ee** coat weight error(g/m^2), **F** ρ widthwise(mΩm), **Fe** ρ w.error(mΩm), **G** ρ lengthwise(mΩm), **Ge** ρ l.error(mΩm), **H** ratio ρ width/length, **He** ratio ρ error, **I** measured sheet resistance (kΩ/sq), **Ie** measured sheet resistance error(kΩ/sq), **J** calculated sheet resistance (kΩ/sq), **Je** calculated sheet resistance error(kΩ/sq), **K** measured capacitance (F/g), **Ke** measured capacitance error (F/g). For sheet resistance two methods was used, either (column **I**) direct measurement by four point probe pushed against coating surface at one hundred locations for each sample or calculated from the bulk resistivity ρ (column **J**). All errors are std. dev. errors.

A	B	C	D	De	E	Ee	F	Fe
roll-01a-55	50	-350	5.24	0.59	0.67	0.31	118	18
roll-01a-29	50	-350	3.84	0.61	0.33	0.31	658	98
roll-01a-49	50	-350	5.01	0.61	0.85	0.30	353	51
sheet-01b	50	-400	12.00	0.71	4.54	0.28	62.2	6.4
sheet-01c	50	-450	9.90	0.66	4.26	0.31	56.4	5.5
sheet-01d	50	-500	10.08	0.72	3.45	0.30	68.1	5.6
sheet-02A	25	-200	6.77	0.61	3.14	0.29	33.9	3.2
sheet-02B	25	-300	7.46	0.68	4.54	0.30	2.42	0.23
sheet-02C	25	-400	16.3	1.2	10.65	0.30	1.72	0.16
roll-UM-55	25	-700	7.61	0.66	4.89	0.31	7.3	1.0
roll-UM-29	25	-700	8.41	0.66	4.07	0.28	11.9	1.6
roll-UM-49	25	-700	8.21	0.84	3.47	0.29	25.7	3.5
roll-03B-55	25	-900	14.33	0.80	7.10	0.30	10.2	1.1
roll-03B-29	25	-900	16.4	1.0	4.39	0.31	48.2	4.3
roll-03B-49	25	-900	13.33	0.80	3.14	0.30	67.3	5.9
roll-06-29	25	-400	25.4	1.1	8.39	0.33	3.88	0.25
roll-07-29	25	-800	46.8	2.1	11.42	0.31	2.22	0.11
roll-08-29	25	-800	25.9	1.1	12.83	0.22	0.483	0.022
roll-10-29	15	-500	38.3	2.8	13.19	0.75	-	-
roll-11-29	20	-400	47.7	1.4	17.65	0.29	1.062	0.037
roll-12-29	25	-400	56.1	2.5	10.79	0.24	9.78	0.45
sheet-cal-06	25	-400	9.99	0.74	8.39	0.33	-	-
sheet-cal-07	25	-800	7.37	0.67	11.42	0.31	-	-
sheet-cal-08	25	-800	25.50	1.11	12.83	0.22	-	-
sheet-cal-11	20	-400	42.30	0.93	17.65	0.29	-	-
sheet-cal-12	25	-400	59.63	1.24	10.79	0.24	-	-

A	G	Ge	H	He	I	Ie	J	Je
roll-01a-55	19.4	2.6	6.07	0.21	10.8	2.00	3.71	0.65
roll-01a-29	86	18	7.66	0.25	81	24	22.4	4.7
roll-01a-49	118	15	2.98	0.19	34	46	23.6	4.1
sheet-01b	17.6	1.1	3.54	0.12	2305	320	1465	124
sheet-01c	26.0	2.0	2.17	0.12	2532	1263	2628	264
sheet-01d	15.1	1.3	4.51	0.12	1450	193	1498	170
sheet-02A	16.8	1.6	2.01	0.14	4188	915	2485	329
sheet-02B	2.01	0.20	1.21	0.14	218	48	269	37
sheet-02C	1.037	0.093	1.66	0.13	56.6	8.1	63.6	7.3
roll-UM-55	2.60	0.28	2.82	0.17	0.217	0.032	0.341	0.047
roll-UM-29	4.53	0.37	2.62	0.16	0.537	0.015	0.539	0.061
roll-UM-49	4.68	0.61	5.49	0.19	0.759	0.091	0.570	0.095
roll-03B-55	2.66	0.22	3.82	0.14	0.196	0.054	0.185	0.018
roll-03B-29	19.5	1.4	2.48	0.11	1.201	0.045	1.19	0.11
roll-03B-49	10.84	0.69	6.21	0.11	1.71	0.75	0.814	0.071
roll-06-29	2.42	0.14	1.604	0.087	-	-	0.0950	0.0070
roll-07-29	1.90	0.10	1.168	0.071	-	-	0.0407	0.0028
roll-08-29	0.355	0.021	1.359	0.076	-	-	0.0137	0.0010
roll-10-29	-	-	-	-	-	-	-	-
roll-11-29	0.958	0.038	1.108	0.053	-	-	0.0201	0.0010
roll-12-29	5.46	0.25	1.790	0.065	-	-	0.0973	0.0062
sheet-cal-06	0.597	0.010	-	-	-	-	-	-
sheet-cal-07	0.193	0.002	-	-	-	-	-	-
sheet-cal-08	0.287	0.004	-	-	-	-	-	-
sheet-cal-11	0.532	0.007	-	-	-	-	-	-
sheet-cal-12	3.661	0.052	-	-	-	-	-	-

7. Electron microscopy study of coating morphology from roll 03B and roll 11

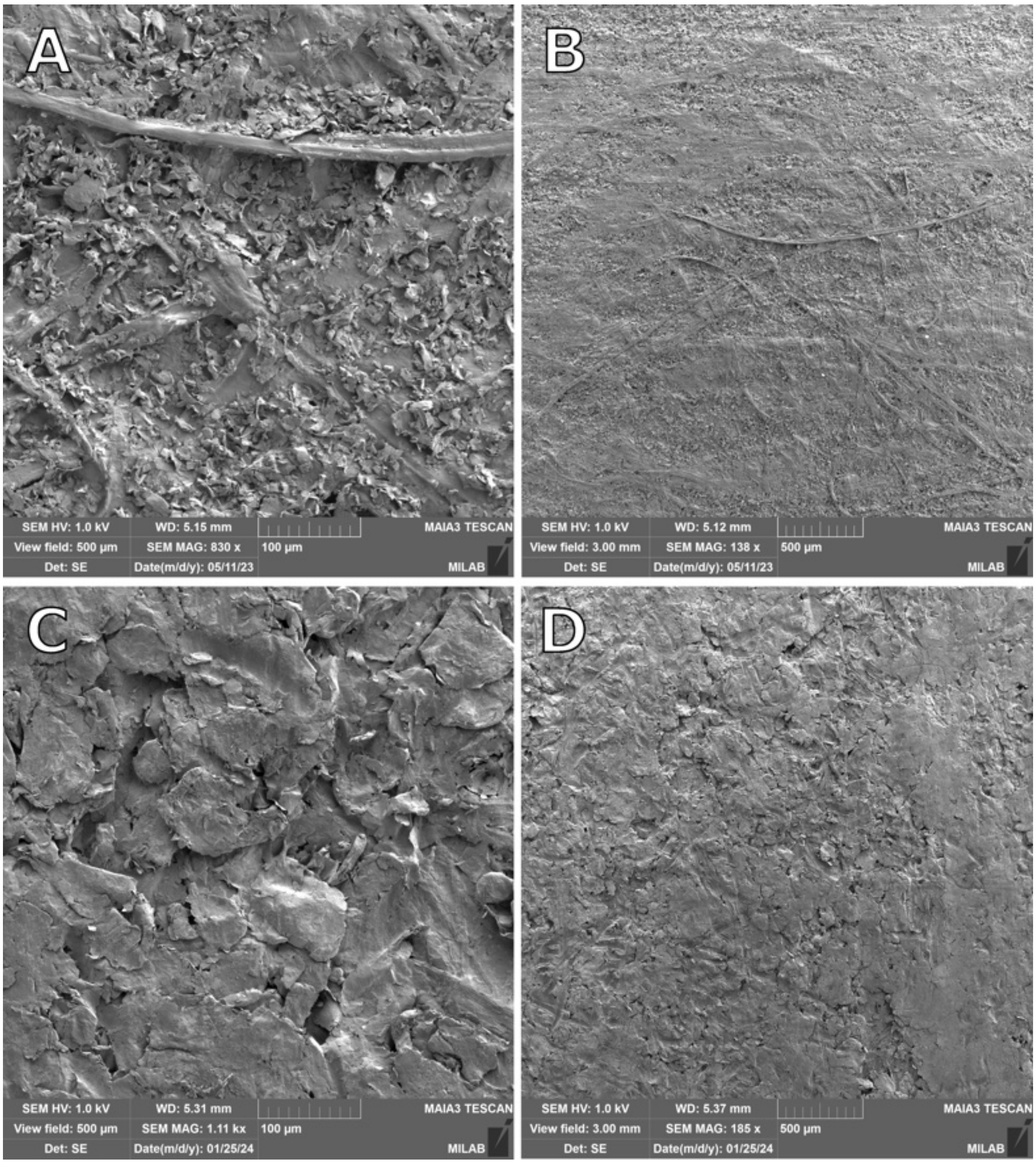

Figure S6. Top row: SEM images from roll 03B (A) detailed top view, (B) lower magnification of top view. Bottom row in similar way but for roll 11, (C) detailed top view, (D) lower magification of top view. Scalebars from left to right both rows: 100 μm and 500 μm. Except for the thinner coating on roll 03B compared to roll 11 the other observations seen are differences in flake size where approximately 10 times smaller flakes are present in slurry A, as seen in the detailed image (A) of roll 03B, compared to (C) made from Slurry B. Another observation is that paper fibers can be seen through the coating at some places (B) compared to (D).

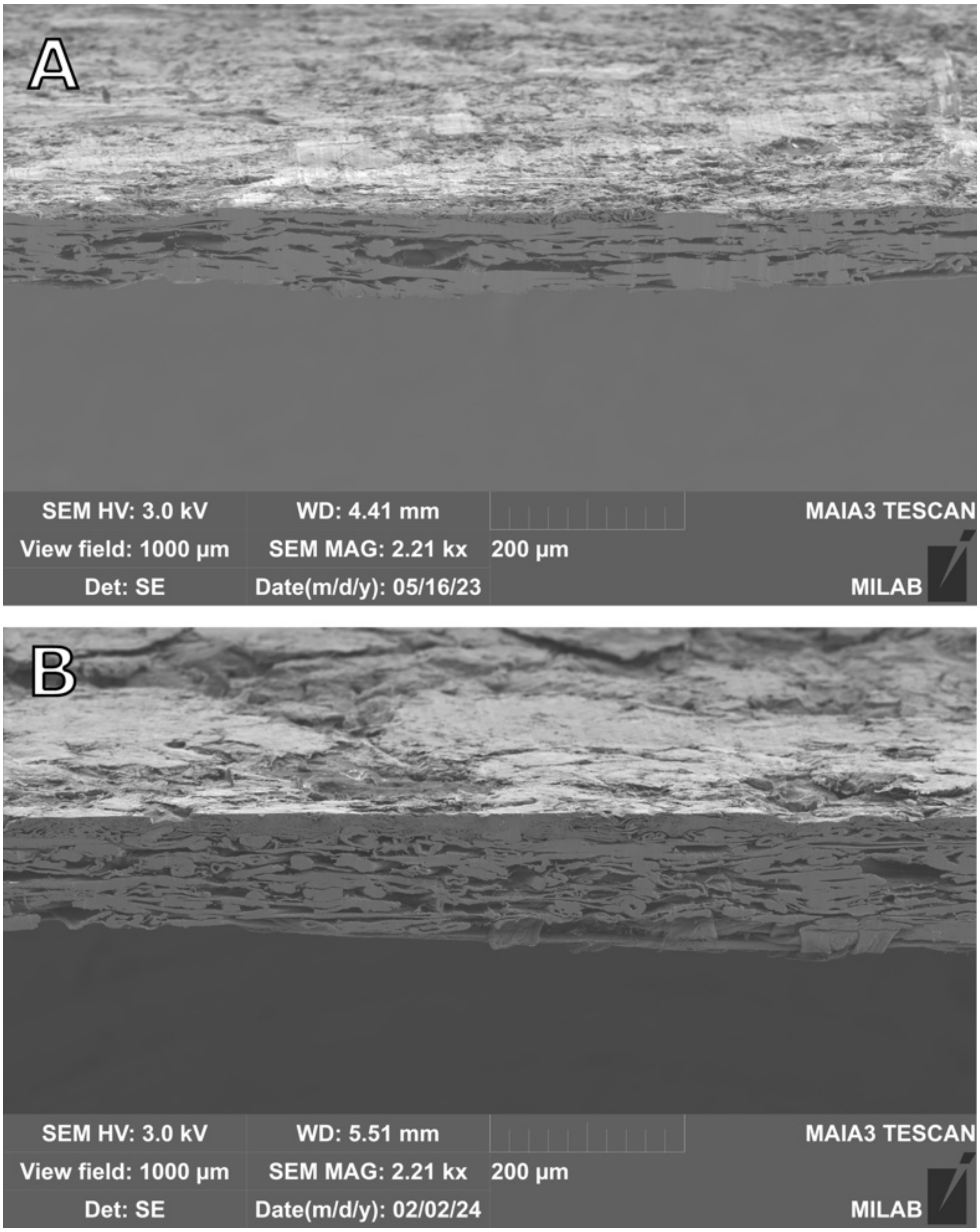

Figure S7. SEM crossectional image in paper lengthwise direction, (A) from roll 03B and (B) from roll 11, scalebars 200 μm. For 03B the coating looks thinner in that the top fibers of the paper are not coated and only the valleys of the paper surface are filled with the nanographite. It also looks somewhat more porous compared to roll 11. Since there is a natural fiber anisotropy of a paper in the favoring alignment of fibers in paper length direction a thin coating that only fills the voids of the paper therefore tends to connect lengthwise at first hand and secondly orthogonal across the fibers, hence leading to anisotropy effects of electrical resistivity with higher resistivity in the width direction of the paper rolls.

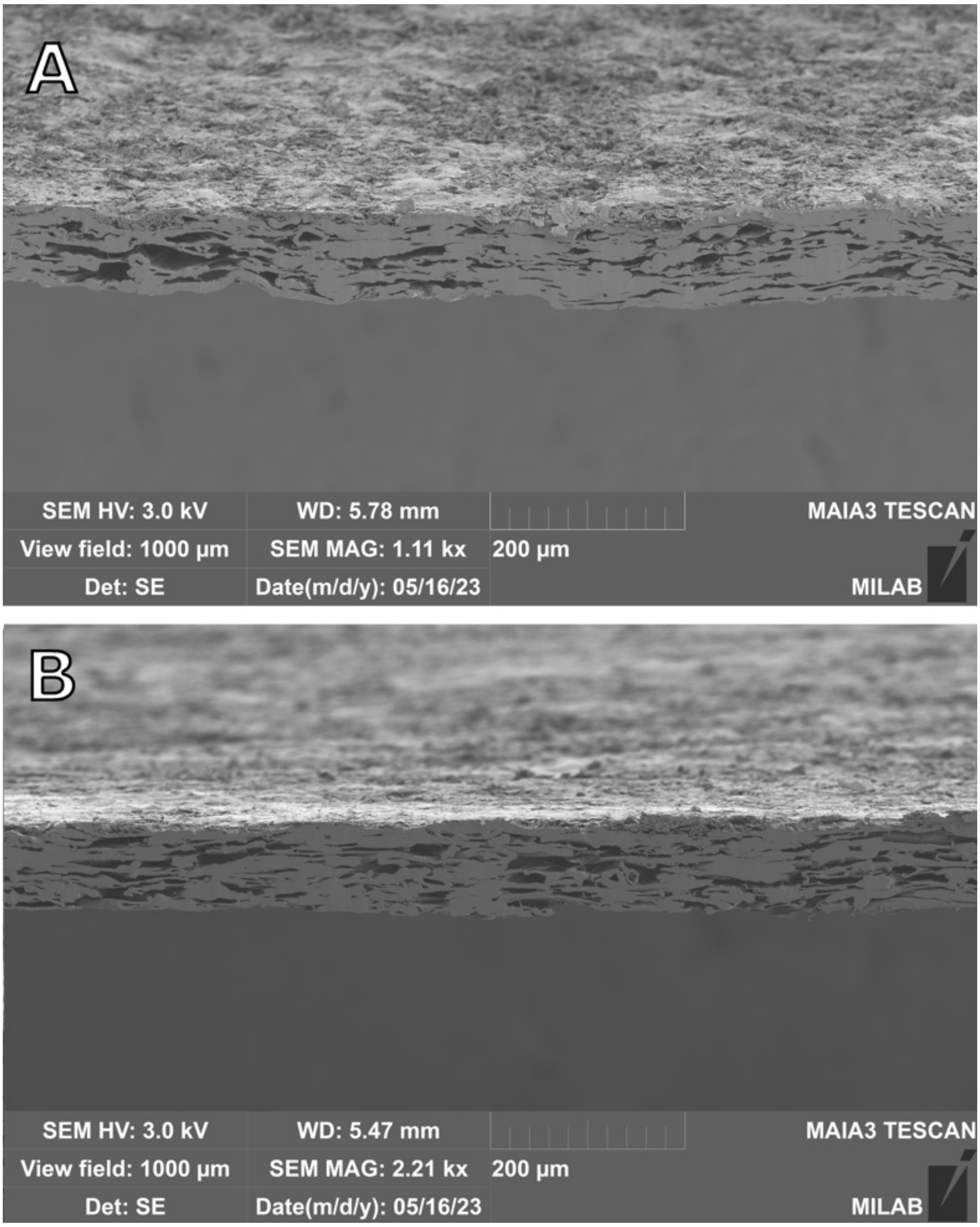

Figure S8. SEM crossectional image in sheets from 02C roll. Image (A) corresponds to crossection taken in the width direction of the roll and (B) the length direction. Scalebars 200 μm. As seen in (A) the surface of the paper is more rough in the horizontal direction of the image compared to image (B) indicating the expected anisotropy of the paper.

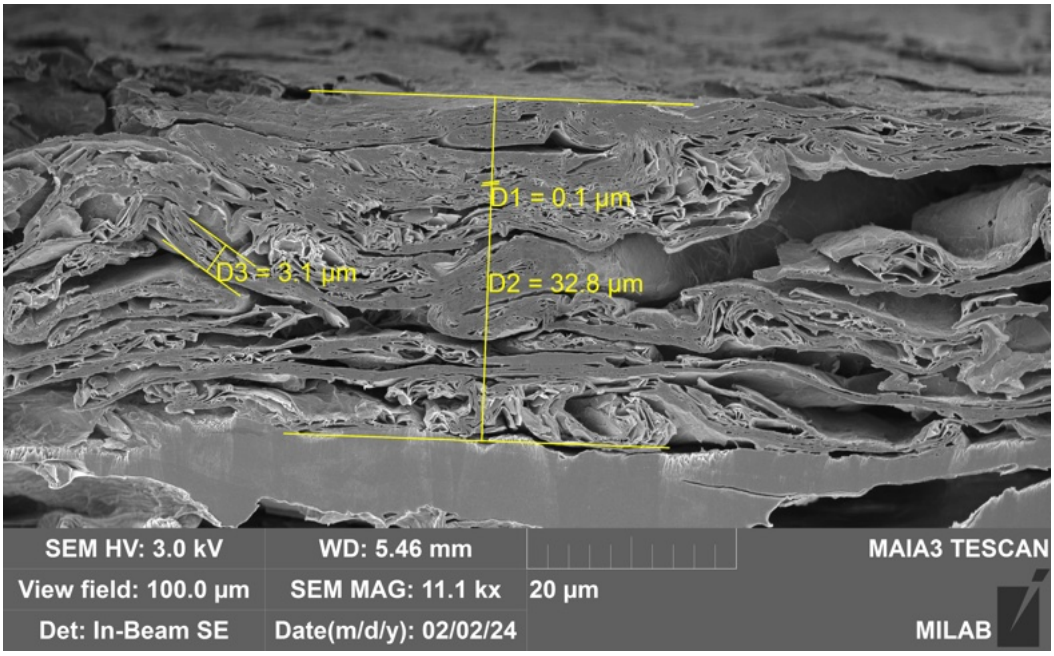

Figure S9. High resolution SEM crossectional image, scalebar 20 μm, from roll 07 showing the nanographite coating layer on top of the paper. Here thickness of coating is measured to be 32.8 μm and the nanographite flakes tend to aggregate into bundles and these are about 3.1 μm in thickness. The thinnest nanographite sections seen in this image is 100 nm. The big solid region at the bottom of image is the top fiber of the paper substrate.

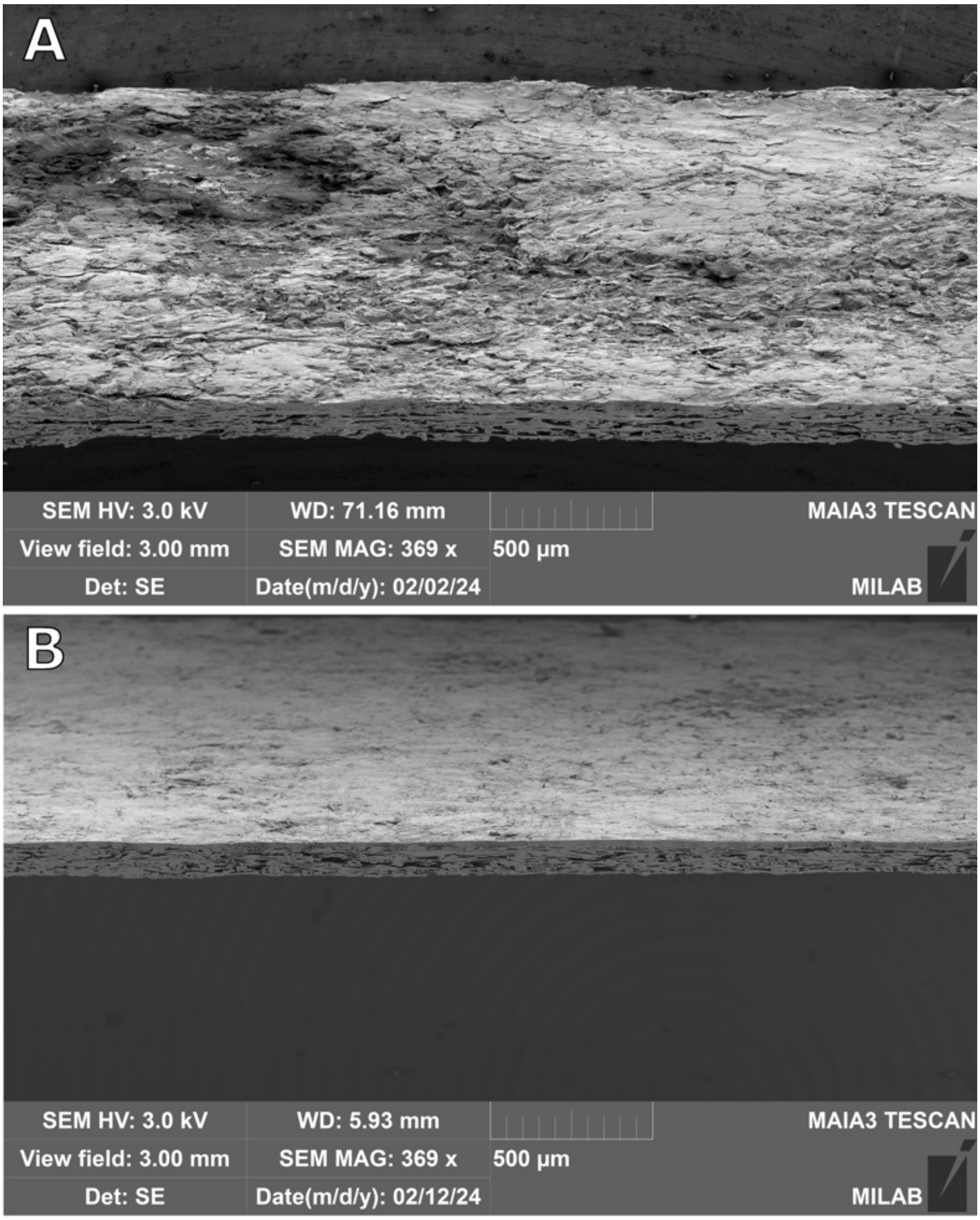

Figure S10. SEM images of crossection as well as the top of coating in perspective, scalebars 500 μm. Image (A) shows roll 12 and the dark regions shows regions with little and in top left of image no coating and with exposed fibers, indicated by the observed charging artifacts in the SEM image. This is seen as one cause of the different behavior of roll 12 in the resistivity measurements in that the lower density coating is not homogenies but rather caused by these larger variations in the coating. Image (B) shows the other end of the spectra the smoothest surface achieved during the pilot trials and is from roll 11 after calendering.

References

1. Blomquist, Nicklas and Alimadadi, Majid and Hummelgård, Magnus and Dahlström, Christina and Olsen, Martin and Olin, Håkan. Effects of geometry on large-scale tube-shear exfoliation of graphite to multilayer graphene and nanographite in water. *Sci. Rep.* **2019**, *9(1)*, 8966.

Disclaimer/Publisher's Note: The statements, opinions and data contained in all publications are solely those of the individual author(s) and contributor(s) and not of MDPI and/or the editor(s). MDPI and/or the editor(s) disclaim responsibility for any injury to people or property resulting from any ideas, methods, instructions or products referred to in the content.
