# Supplementary material for: Large-Scale Compatible Roll-to-Roll Coating of Paper Electrodes and Their Compatibility as Lithium-Ion Battery Anodes
Source: Nanomaterials (Basel). 2025 Jan 14;15(2):113. doi: 10.3390/nano15020113 (PMC11767952; doi:10.3390/nano15020113)
Supplement: Supplementary file 1 [file nanomaterials-15-00113-s001.zip › Supplementary- Large-scale/S4-graph-03B-UM-rolls-anisotrophic-resistivity-lengthwise-shelflife.pdf]

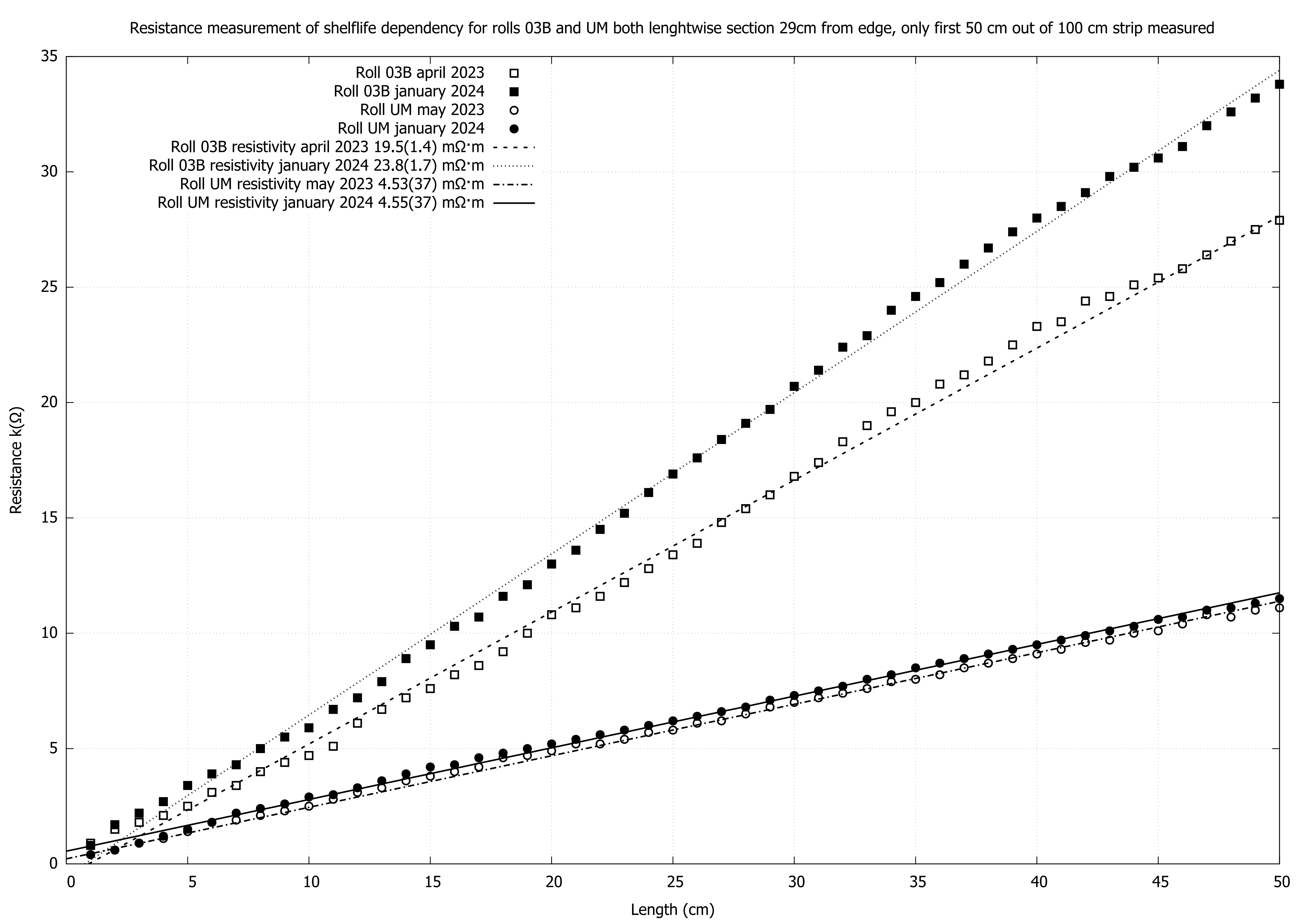
Resistance measurement of shelflife dependency for rolls 03B and UM both lenghtwise section 29cm from edge, only first 50 cm out of 100 cm strip measured
Roll 03B april 2023
Roll 03B january 2024
Roll UM may 2023
Roll UM january 2024
Roll 03B resistivity april 2023 19.5(1.4) mΩ·m
Roll 03B resistivity january 2024 23.8(1.7) mΩ·m
Roll UM resistivity may 2023 4.53(37) mΩ·m
Roll UM resistivity january 2024 4.55(37) mΩ·m
Resistance k(Ω)
Length (cm)
0
5
10
15
20
25
30
35
40
45
50
