# Supplementary figures and images for: Large-Scale Compatible Roll-to-Roll Coating of Paper Electrodes and Their Compatibility as Lithium-Ion Battery Anodes
Source: Nanomaterials (Basel). 2025 Jan 14;15(2):113. doi: 10.3390/nano15020113 (PMC11767952; doi:10.3390/nano15020113)

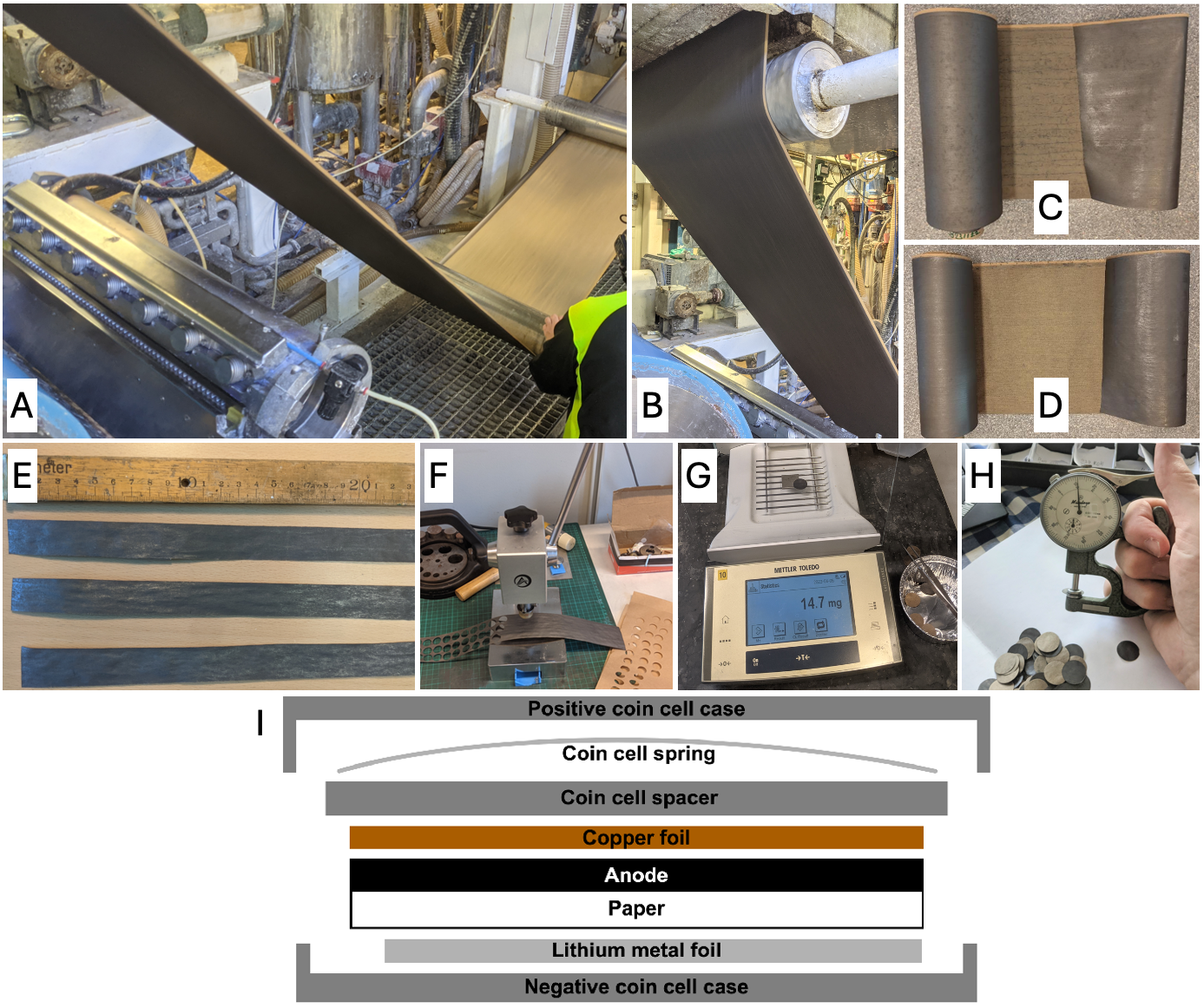

Supplement: Supplementary file 1 [file nanomaterials-15-00113-s001.zip › Supplementary- Large-scale/S2-coat.png]

Resistivity as function of relative humidity in O2 B,C sheets

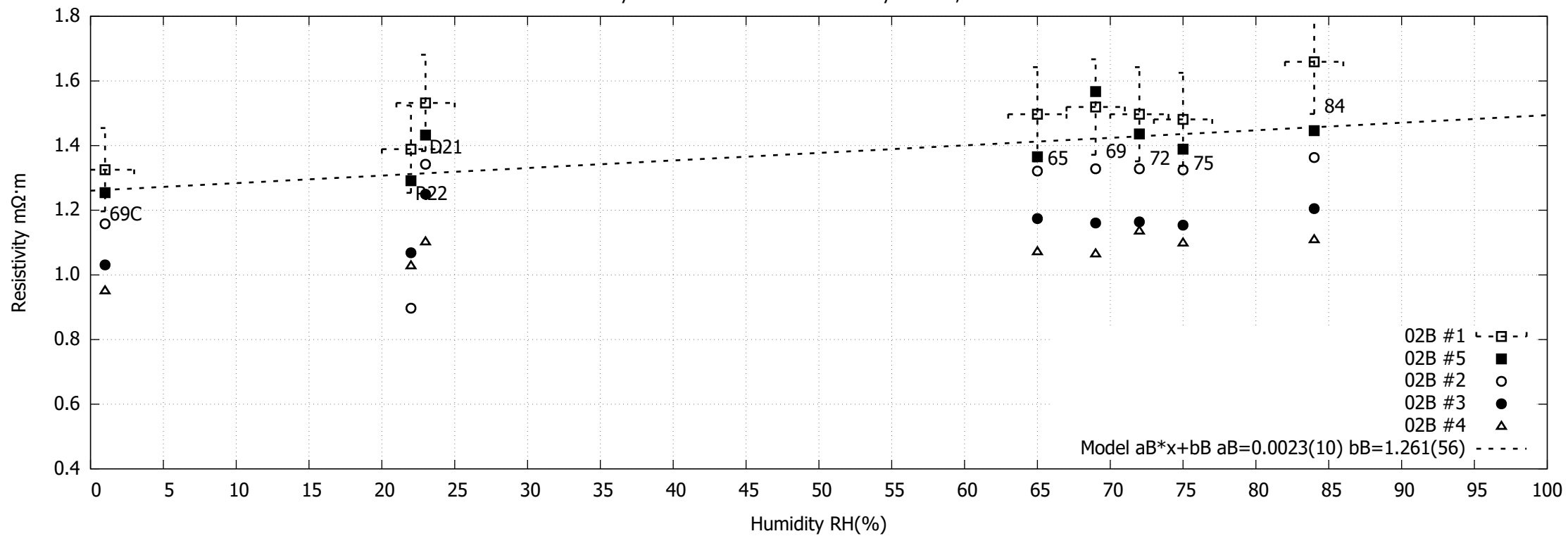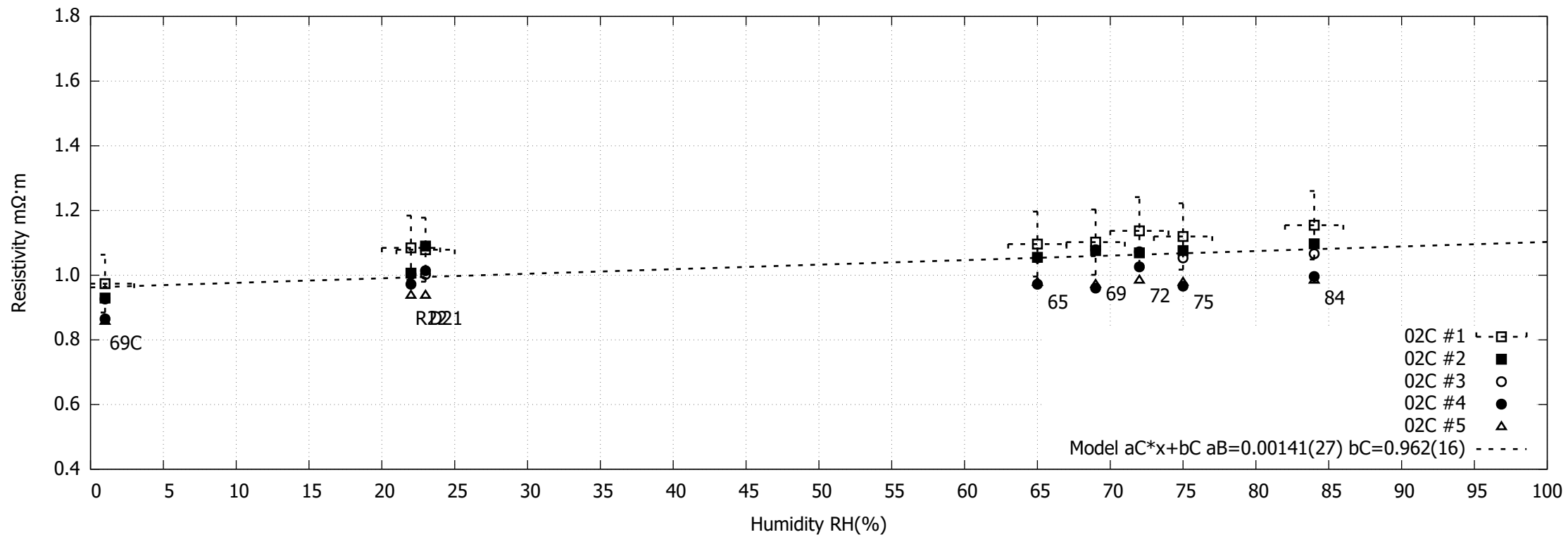

Supplement: Supplementary file 1 [file nanomaterials-15-00113-s001.zip › Supplementary- Large-scale/S3-graph-02BC-sheets-resistivity-humidity.pdf]

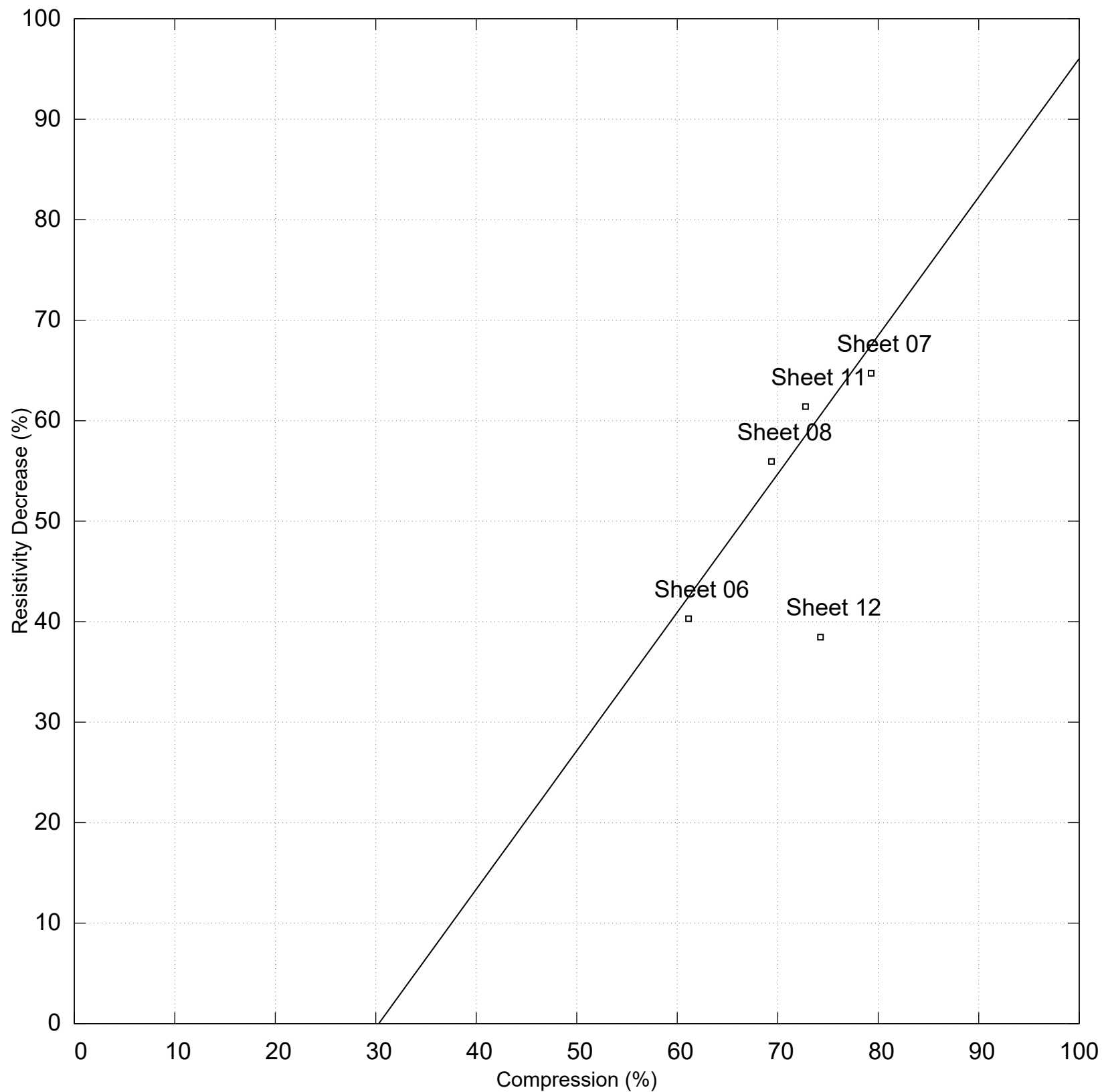

Supplement: Supplementary file 1 [file nanomaterials-15-00113-s001.zip › Supplementary- Large-scale/S5-graph-sheets-resistivity-compression-PUBLICATION.pdf]

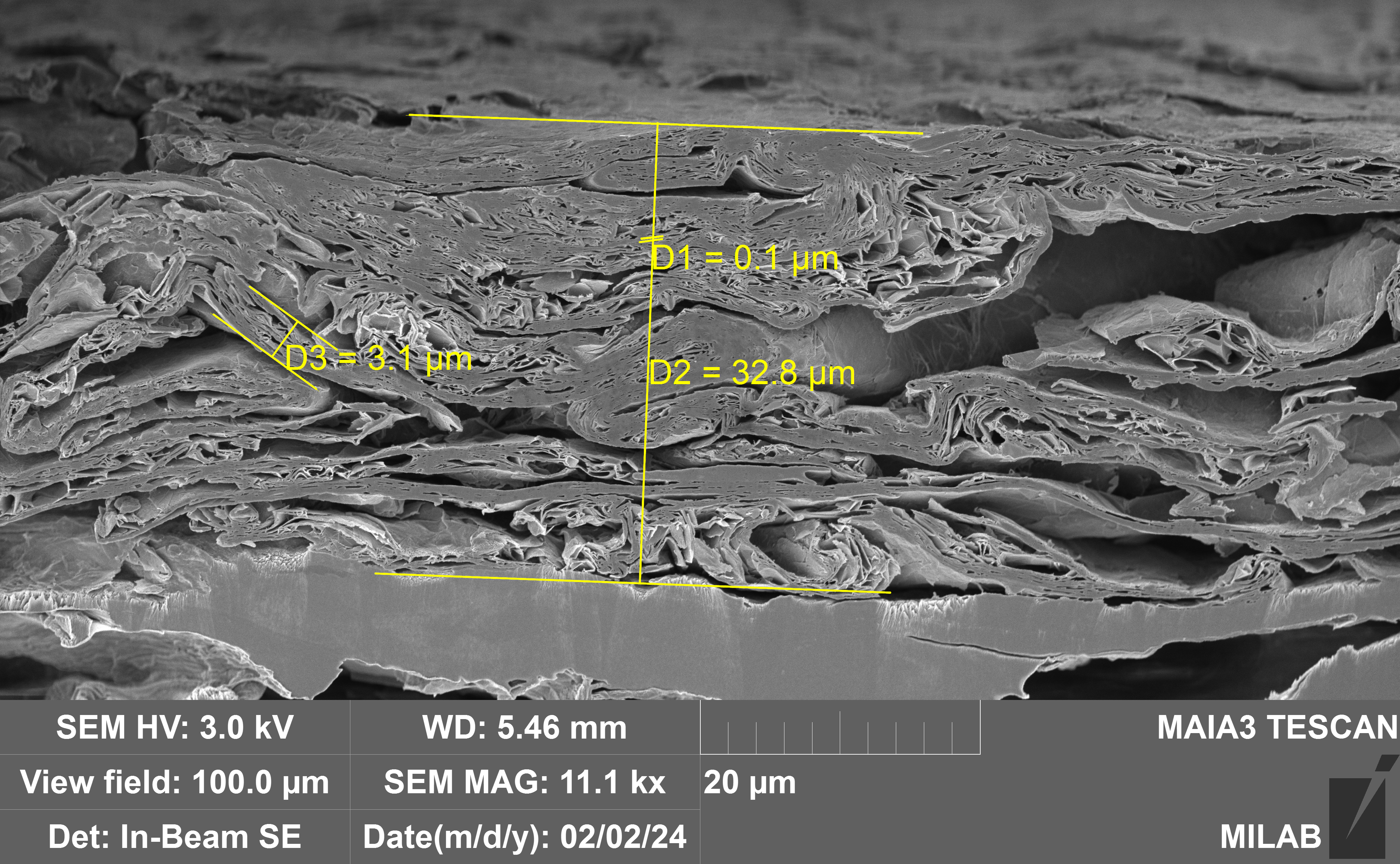

Supplement: Supplementary file 1 [file nanomaterials-15-00113-s001.zip › Supplementary- Large-scale/S9-roll07-inbeamSE-100um-3kv-01m.png]

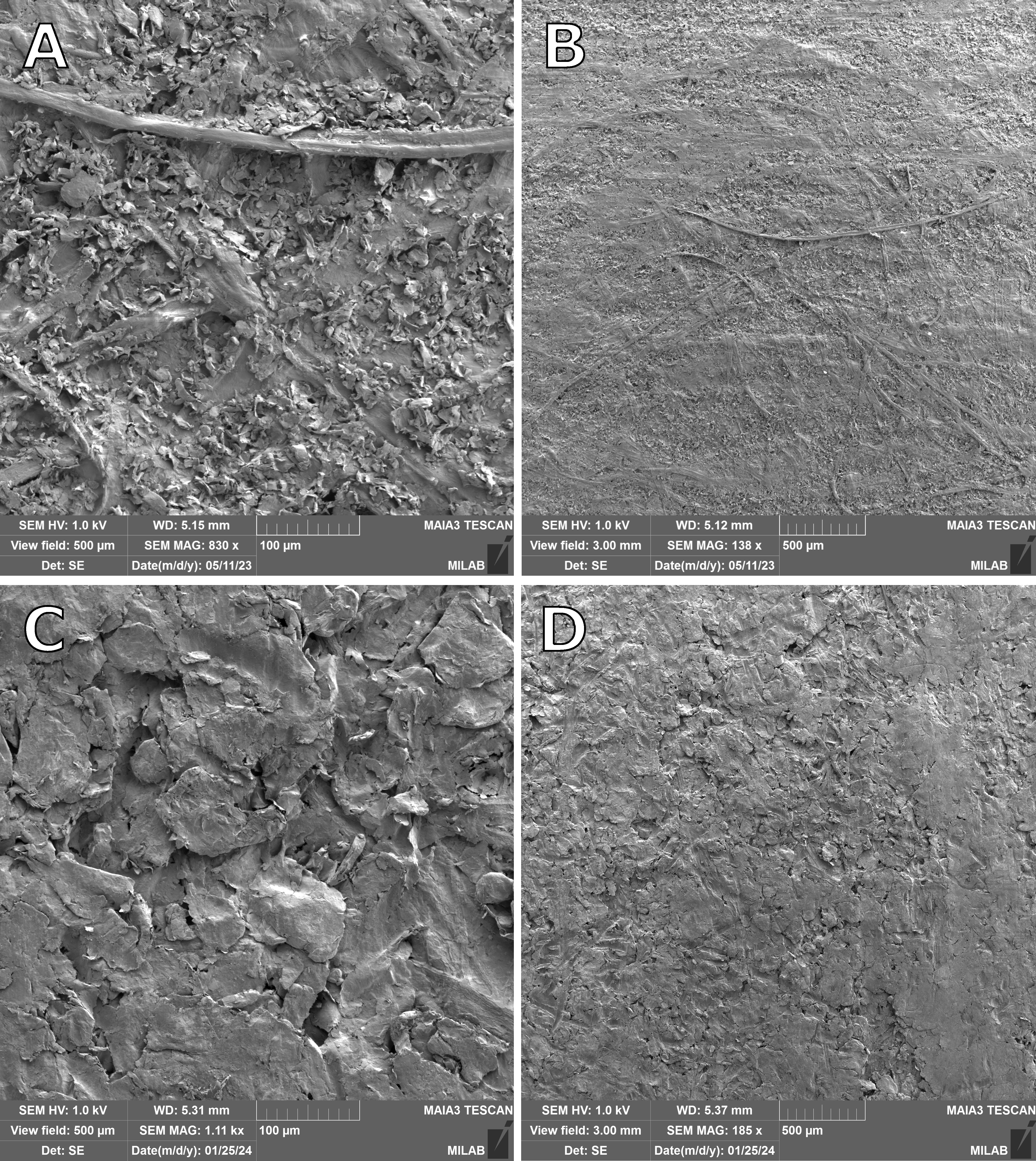

Supplement: Supplementary file 1 [file nanomaterials-15-00113-s001.zip › Supplementary- Large-scale/S6-03B-11-SEM-overview.png]

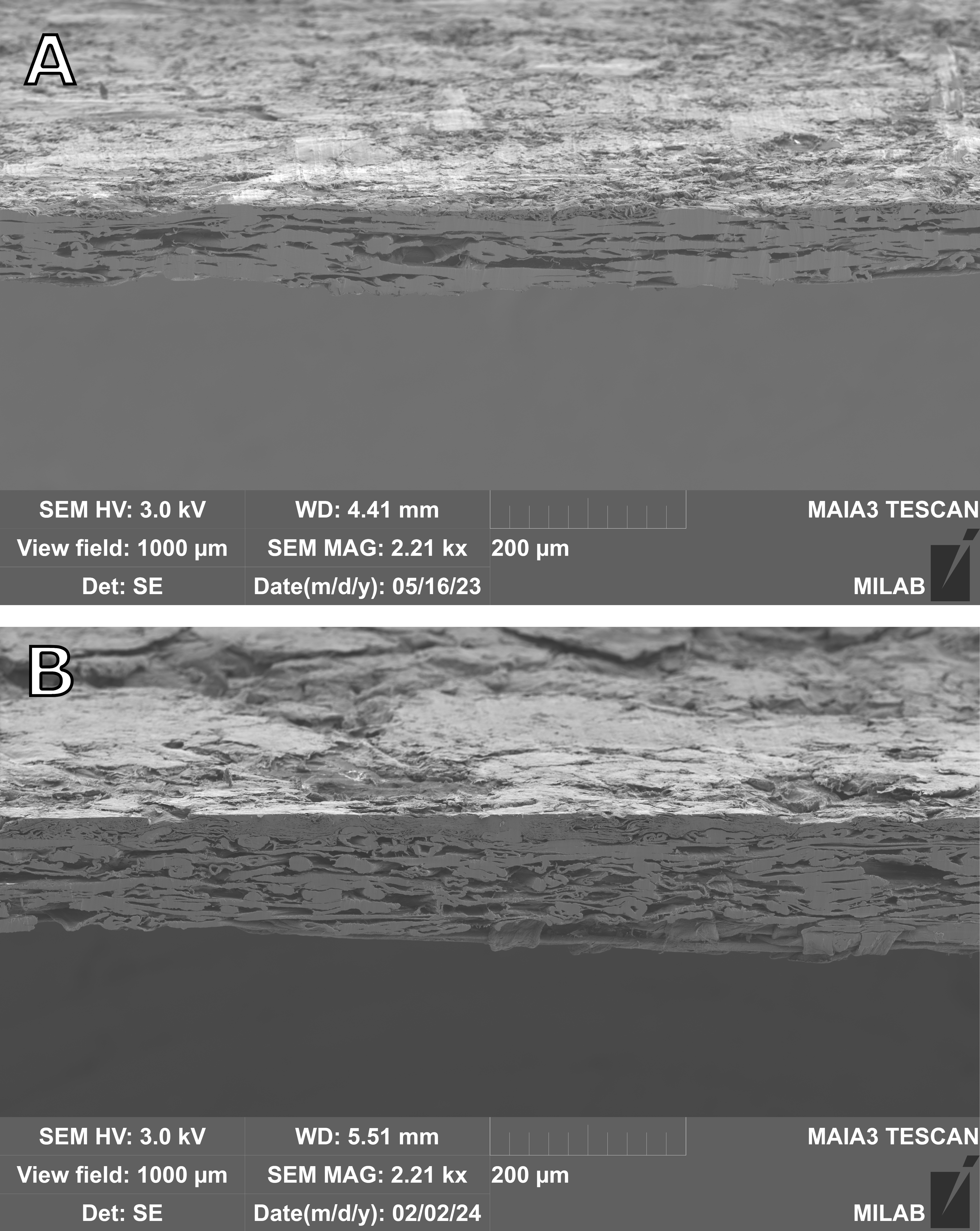

Supplement: Supplementary file 1 [file nanomaterials-15-00113-s001.zip › Supplementary- Large-scale/S7-03B-11-crosscut.png]

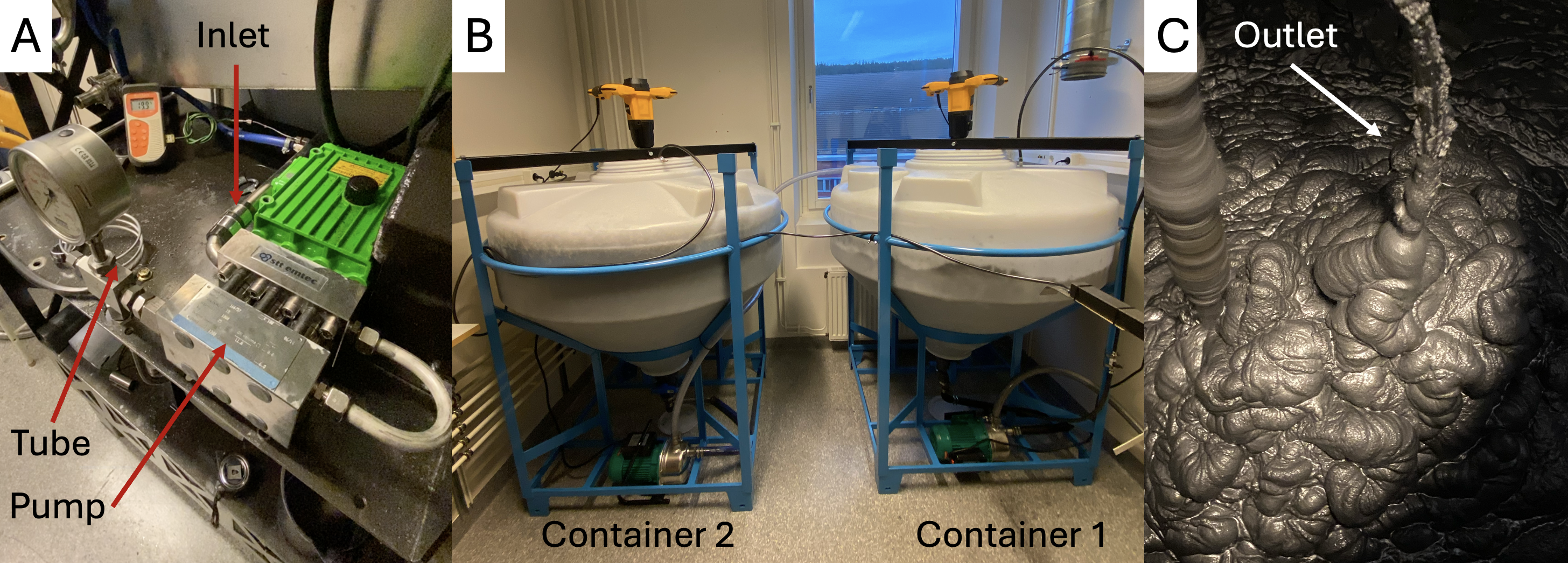

Supplement: Supplementary file 1 [file nanomaterials-15-00113-s001.zip › Supplementary- Large-scale/S1-exfoliation.png]

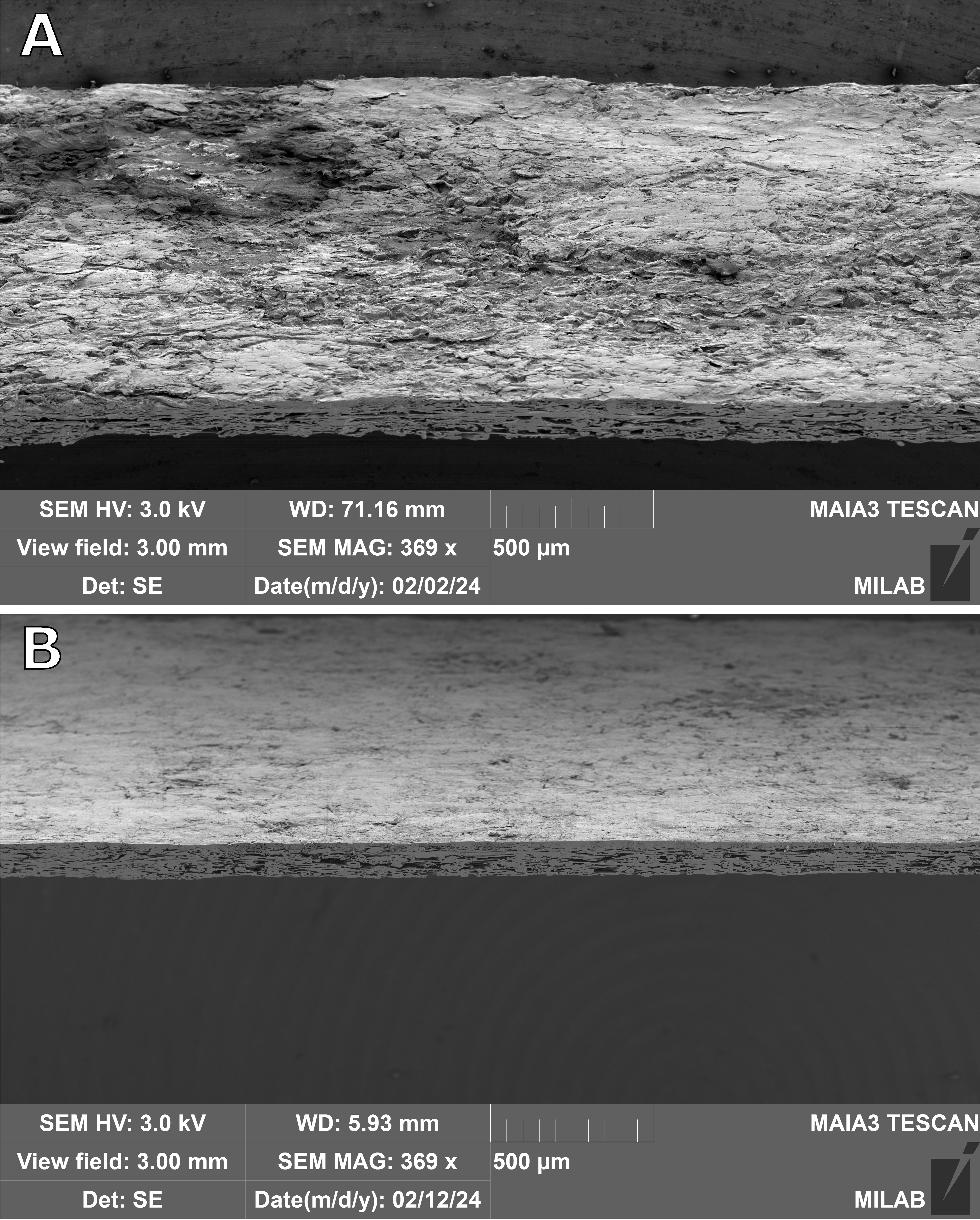

Supplement: Supplementary file 1 [file nanomaterials-15-00113-s001.zip › Supplementary- Large-scale/S10-perspective-roll12-roll11cal.png]

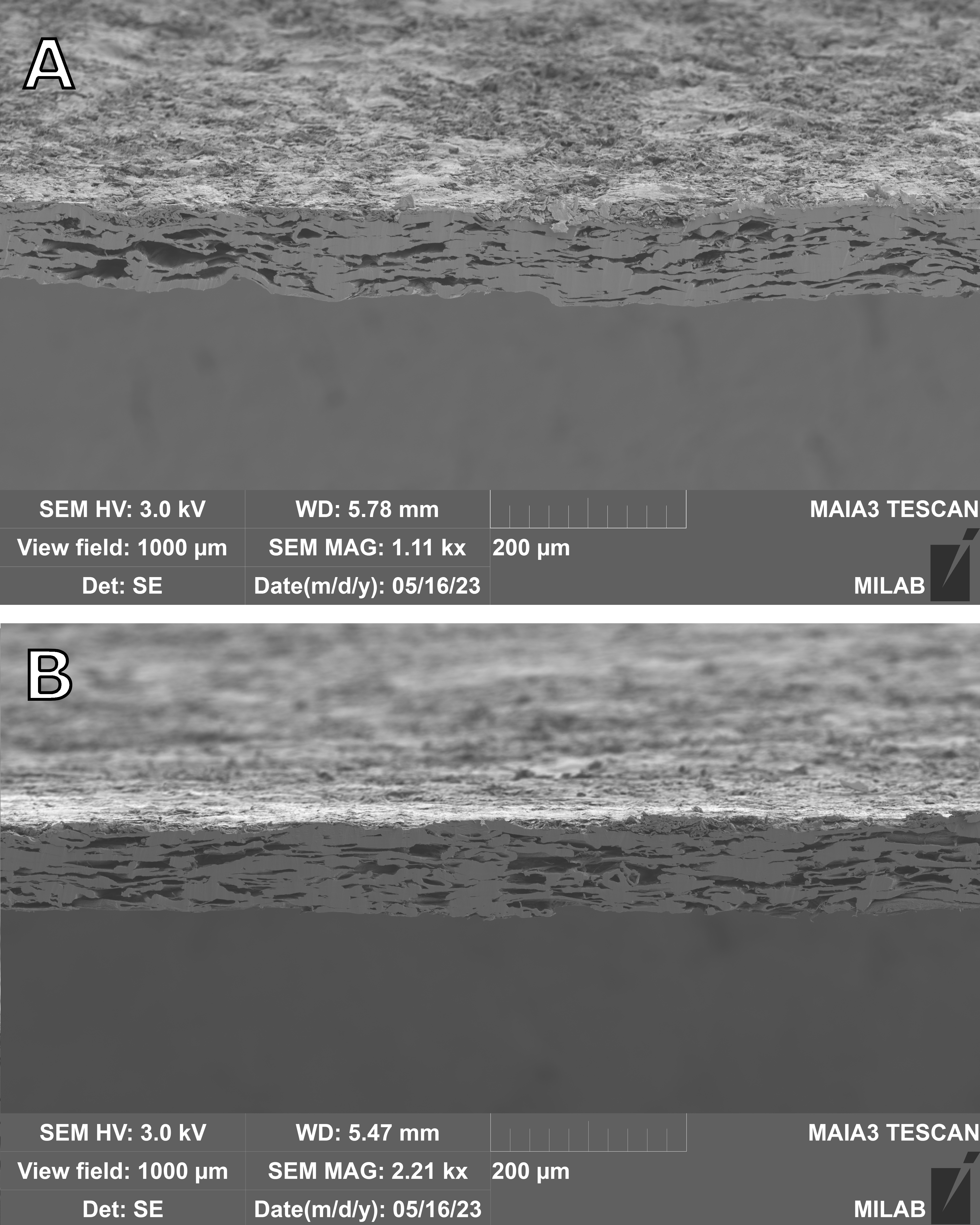

Supplement: Supplementary file 1 [file nanomaterials-15-00113-s001.zip › Supplementary- Large-scale/S8-02C-width-length-crosscut.png]

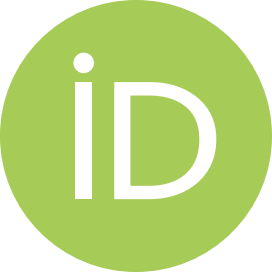

Supplement: Supplementary file 1 [file nanomaterials-15-00113-s001.zip › Supplementary- Large-scale/Definitions/logo-orcid.pdf]
